# Characterization of mineral phosphate solubilization traits from a barley rhizosphere soil functional metagenome

**DOI:** 10.1002/mbo3.110

**Published:** 2013-07-25

**Authors:** Sagar Chhabra, Dina Brazil, John Morrissey, James I Burke, Fergal O'Gara, David N Dowling

**Affiliations:** 1 Department of Science and Health, Institute of Technology CarlowCarlow, Ireland; 2 Department of Microbiology, University College CorkCork, Ireland; 3 National Plant Biotechnology Centre, Teagasc OakParkCarlow, Ireland

**Keywords:** Glucose dehydrogenase, MPS, pyrroloquinoline quinone, soil metagenome.

## Abstract

Mineral phosphate solubilization (MPS) microorganisms are important for their provision of orthophosphate anions for plant growth promotion activity in soil. In this study, we applied a functional metagenomic approach to identify this trait directly from the microbiome in barley rhizosphere soil that had not received P fertilizer over a 15-year period. A fosmid system was used to clone the metagenome of which 18,000 clones (∼666 Mb of DNA) was screened for MPS. Functional assays and High Performance Liquid Chromatography analysis recognized gluconic acid production and MPS activity in the range 24.8–77.1 mmol/L and 27.6–38.16 μg/mL, respectively, when screened in an *Escherichia coli* host (at frequency of one MPS-positive clone hit per 114 Mb DNA tested). The MPS clones (with average insert size of ∼37 kb) were analysed by 454 Roche sequencing and annotated. A number of genes/operons with homology to Phosphorous (P) uptake, regulatory and solubilization mechanisms were identified, linking the MPS function to the uncultivated microbiome present in barley rhizosphere soil.

## Introduction

Phosphorous (P) is the most applied fertilizer in soil next to nitrogen in crop production (Goldstein 2007). However, availability of P (in a plant available form) remains low in many farming systems (Stevenson 1986; Goldstein 2007). Phosphate-solubilizing microorganisms can be a useful biofertilizer resource to supply the plant available form of P (as orthophosphate anions) and therefore are frequently explored for P solubilizing potential (Sashidhar and Podile 2010). Several studies have recognized the genes involved in mineral phosphate solubilization (MPS) activity from bacterial isolates by cloning and complementation of MPS traits in a bacterial host system such as *Escherichia coli*. (Goldstein and Liu 1987; Babu-Khan et al. 1995; Kim et al. 1997, 1998). *E. coli* itself lacks the necessary genes required for P solubilization activity such as pyrroloquinoline quinone (PQQ) synthesis (Liu et al. 1992; Krishnaraj and Goldstein 2001). The genetic basis/mechanism of MPS is not completely elucidated (Rodriguez and Fraga 1999), however, it is understood that gluconic acid (GA) and 2-ketogluconic acid (2-KGA) biosynthesis in the periplasm of bacteria (direct oxidation pathway) can be an important basis for MPS activity in many Gram-negative bacteria (Sashidhar and Podile 2010). GA biosynthesis is commonly carried out by the enzyme glucose dehydrogenase (GCD) in the presence of the cofactor, PQQ (Shen et al. 2012). Alternative genes involved in GA production have also been identified, for example, a *gabY* gene cloned from *Pseudomonas cepacia* in *E. coli* was shown to be involved in MPS activity. The deduced amino acid sequence of this gene was shown to have no similarity to the commonly known GA biosynthesis genes (PQQ or GCD), but showed homology to histidine permease membrane-bound components (Babu-Khan et al. 1995). In addition, a DNA fragment from *Serratia marcescens* that induces GA synthesis in *E. coli* was identified which showed no homology to PQQ or GCD genes (Krishnaraj and Goldstein 2001).

In this study, we utilized a functional metagenomic and sequencing approach in an attempt to identify/characterize the MPS trait directly from the microbiome of barley rhizosphere soil. The physiological potential activity of the MPS clones was characterized using functional assays while the MPS clones (with an average insert size ∼37 kb) were sequenced to relate the MPS activity to potential gene(s) involved in P solubilization.

## Materials and Methods

### Soil sample collection

The experimental Knockbeg field site is located at Teagasc Oakpark Crop Research Center, Carlow, Ireland (52°51′N, 6°56′W). The soil is a deep (>1 m) medium-heavy textured, free-draining gray-brown podzolic soil type derived from limestone boulder clay (Knockbeg series) with an average organic matter of 5% (Fay and Zhang 2012). The sampled plots measure 12.5 × 30 m and are continuously cropped to spring barley monocultures since 1994. Sampling was carried out from the rhizosphere soil of barley grown under a low-input mineral management regime. The details of mineral management practices in these sites have been mentioned previously (Conry and Hogan 2001; Chhabra et al. 2013). Sampling from this site was carried out in the last week of February 2010; the experimental design for sampling was a randomized complete factorial. Ten randomly chosen plants per plot were removed and the adhering soil was placed in sterile plastic bags from four replicate plots of barley, after removal of roots the soil samples were homogenized and were pooled together in order to obtain a representative sample for library construction. The samples were transported on ice and stored at 4°C before extraction of high-molecular weight (HMW) DNA.

### DNA isolation and fosmid library construction

High-molecular weight DNA extraction was carried out using the Brady (2007) protocol. The metagenomic library was constructed in the pCC1FOS vector with the CopyControl™ fosmid library system according to the manufacturer's instructions (Epicentre, Madison, WI). Briefly, purified metagenomic DNA was size selected (i.e., with an average size of 37 Kb) from a pulsed-field agarose gel run for 16 h (with settings ramp rate 6V/cm; angle 120°;buffer temperature 14°C; internal switch time settings 1–25) before ligation into the pCC1FOS vector. Ligated DNA was then packaged (MaxPlax Lambda Packaging Extract) and titered onto the EPI300-T1^R^ cloning strain (Epicentre, Madison, WI) and plated onto Luria–Bertani containing 12.5 μg/mL chloramphenicol for the selection of recombinant clones. To induce the production of higher fosmid copy numbers, arabinose (0.01% w/v) was added to the media with the targeted clones. The recombinant *E. coli* clones were robotically picked using a QPix (Molecular Devices, Sunnyvale, CA) colony picker at the Environmental Research Institute (ERI) in University College Cork (UCC). The library was replicated onto 384-well plates containing Luria–Bertani broth supplemented with chloramphenicol (12.5 μg/mL).

### Screening and MPS activity determination

To test for MPS activity (i.e., Ca_3_(PO_4_)_2_ solubilization) a sample of the library (18,000 out of 60,000 clones) was plated on National Botanic Research Institute Phosphate (NBRIP) agar plates (Nautiyal 1999) containing 12.5 μg/mL chloramphenicol. The plates were incubated for 4 days at 37°C to detect MPS activity (i.e., halo formation around the colony on the plate). The MPS-positive clones (6) from the library were retested by growing individual clones overnight at 37°C in 100 mL LB-broth in a conical flask. The inoculum (3 μL with similar OD ∼1) was replated in duplicates on NBRIP agar plates supplemented with 25 μg/mL bromophenol blue (BPB) and 12.5 μg/mL chloramphenicol under same conditions as mentioned before. *E. coli* (EPI300-T1^R^) served as a negative control. Quantitative estimation of phosphate solubilization in broths was estimated using the Fiske and Subbarow (1925) method. An overnight cultured LB-broth with individual clones (1 mL with OD ∼1) was inoculated in 100 mL of NBRIP media and was incubated in duplicate at 37°C for 4 days in an orbital shaking incubator set to 180 rpm. Estimation of available P in broth was carried out by withdrawing 100 μL of supernatant from 1 mL culture after centrifugation of broth at 8,000 rpm for 10 min. The pH changes due to the extracellular activity of the MPS clones and also controls (*E. coli* EPI300-T1^R^ and the uninoculated broth) were measured by taking aliquots (5 mL) of cultured NBRIP broth over a period of 4 days and the pH was measured using the pH electrode (Bench pH meter 3510, Jenway, U.K.). The pH of the NBRIP broth was adjusted to pH 7.0 before inoculation/incubation of cells in the broth. High Performance Liquid Chromatography (HPLC) analysis was carried out using a Shimadzu Prominence HPLC (Shimadzu, Japan) using a standard C18 column after incubation of NBRIP broth as mentioned before. Approximately 500 μL of the supernatant was withdrawn from NBRIP cultured broth and filter sterilized (0.2 μm) and used for GA and 2-KGA determination at 210 nm. To estimate insert size of MPS positive clones, restriction fragment length polymorphism (RFLP) profiles were generated using the restriction enzyme *Bam*HI (Promega, Madison, WI). The resulting digest reactions were electrophoresed on an agarose gel 0.8% (w/v) in 1× Tris-Acetate-EDTA buffer at 45 V for 18 h.

### DNA Sequencing and Sequence Annotation

DNA 454 sequencing was carried out by the Centre for Genomic Research at University of Liverpool (U.K.). Full length fosmids samples (6) were sequenced (average insert size ∼37 kb) by preparing six barcoded fragment libraries and sequenced as a multiplexed pool. The sequence data were assembled using the Newbler program version 2.3 (Roche, Basel, Switzerland) and was analyzed and annotated during the course of this study. The gene calling of sequenced data was carried out using the Fgenesb annotator pipeline (Softberry Inc., Mount Kisco, NY) (http://linux1.softberry.com), GeneMark.hmm prediction tool (http://exon.gatech.edu/genemark/) (Besemer and Borodovsky 1999) and ORF Finder (open reading frame finder) (http://www.ncbi.nlm.nih.gov/) (Wheeler et al. 2003). Homology searches for deduced proteins were performed by searching against the nonredundant database, sourced from the nucleotide (nr/nt) collection using the BLAST program (Ye et al. 2006). The annotation of contigs was carried out using an online trial version of CLC DNA workbench (http://www.clcbio.com/index.php). The contigs containing P-associated reading frames generated during this study have been deposited into the Genbank database under accession numbers JQ970523-JQ970528.

## Results

### Identification and isolation of MPS active clones in *E. coli*

The barley rhizosphere soil metagenome had an estimated ∼2220 MB of DNA. (60,000 fosmid clones with estimated average insert size ∼37 kb). A sample of 18,000 colonies were screened for MPS activity and six different MPS positive clones were identified using the NBRIP media plate assay with a frequency of one MPS-positive clone per estimated 114 Mb of DNA tested.

### MPS plate and broth assays

**Figure 1:**
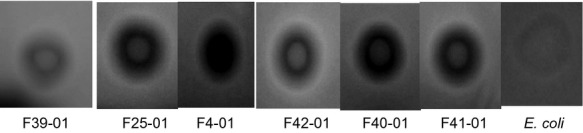
BPB-based plate assay showing extracellular activity of *Escherichia coli* (EPI300-T1^R^) with and without recombinants plasmids.

The inorganic phosphate solubilization abilities of six MPS-positive clones in the library were compared using NBRIP medium modified with BPB (Gupta et al. 1994) to evaluate the level of phosphate solubilization activity. The plates incubated with individual clones showed slight differences in activity (i.e., halo formation around the colony Fig. 1) in comparison to the *E. coli* negative control. To confirm P solubilization activity, the available P level in the NBRIP broths was measured from the MPS clones (in *E. coli* EPI300-T1^R^) after incubation over a period of 4 days. The MPS clone F42-01 produced the highest amount of available P in broth with 38.1 μg/mL of Pi (inorganic phosphate), while clone F4-01 gave the least amount of available P in the broth with 27.6 μg/mL of available P (see Table 1 for more details).

**Table 1:** Broth and HPLC analysis of P solubilizer recombinants encoded in *Escherichia coli* (EPI300-T1^R^)

Fosmid	pH	Available P (μg/mL)	Gluconic acid (mmol/L)
F39-01	4.81 ± 0.07	33.28 ± 0.16	32.2 ± 0.91
F25-01	4.35 ± 0.20	35.1 ± 0.6	35.17 ± 0.09
F4-01	4.77 ± 0.01	27.5 ± 0.55	30.12 ± 0.05
F42-01	4.50 ± 0.10	38.1 ± 0.91	77.13 ± 1.56
F40-01	4.79 ± 0.02	30.5 ± 1.75	24.8 ± 6.39
F41-01	4.59 ± 0.07	32.2 ± 2.12	37.1 ± 0.70
*E. coli*^2^	5.98 ± 0.08	5.7 ± 2.2	NPR^1^
Blank	6.42 ± 0.0	4.05 ± 1.2	NPR^1^

1 NPR, no peak recognized.

2 *E. coli*, negative control no fosmid.

### MPS Fosmids can acidify the medium and confer GA production

To determine extracellular activity (acidification) conferred by the MPS clones, the pH changes of cultured inoculated NBRIP broths (in duplicate) were measured for a period of 4 days. The rate of pH drop was highest during day 1 of incubation, with a pH fall from 7 to a pH range of 5.1–4.75 depending on the clone. MPS clone F40-01 and F39-01 had the highest pH (5.1) whereas clone F25-01 had the lowest pH (4.75). There was a subsequent fall in the level of pH each day in each cultured broth. The overall pH change on day 4 between the MPS clones was in the range between 4.81 and 4.35. The MPS clone F39-01 had the highest pH (4.81) while clone F25-01 had the lowest pH (4.35).

To relate P solubilization activity to organic acid production, HPLC analysis was carried out, on cultured broths incubated over a period of 4 days. The results from HPLC analysis detected a standard GA retention peak at 3.33 min for most of the MPS-positive clones. MPS clone F42-01 had highest GA production in broth with a concentration of 77.1 mmol/L, while the clone F40-01 produced the lowest concentration of GA 24.8 mmol/L in the broth. The range of GA produced by the other clones was between 32.2 mmol/L and 38.1 mmol/L (see Table 1).

### DNA sequence and annotation of Fosmid clones show P solubilization, regulatory, transport and related functions

**Figure 2:**
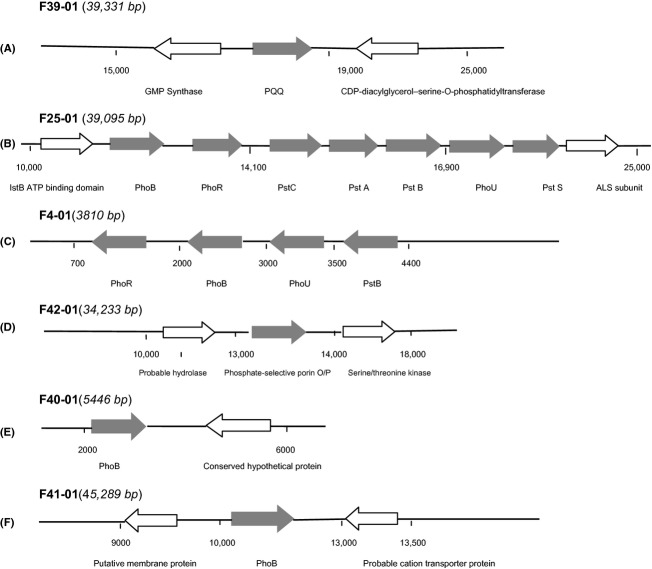
Schematic A–F shows P-associated open reading frames (ORFs) on contigs and adjoining regions (arrows without colour); the full contigs were submitted to Genbank under accession no. JQ970523-JQ970528. The scale (bp) shows the associated regions on the contigs.

The sequence data were analyzed and annotated using gene calling tools (see Fig. 2 and Table 2 for details). Figure 2A indicates functional regions associated with the PQQ system associated with MPS activity. *E. coli* is known to synthesize apo-GCD, but lacks the cofactor PQQ, which is required for P solubilization activity (Liu et al. 1992). Figure 2B–F shows the annotation of partial or full regulatory networks associated with the Pho regulon. The Pho regulon is mainly associated with P regulation activity in bacteria. These genes are controlled by inorganic phosphate (Pi) and are regulated by PhoB (Torriani-Gorini 1994). The P-associated reading frames from MPS clone F25-01 (Fig. 2B) are homologous to a complete Pho regulon with homology of reading frames to sensor kinases (PhoR), the regulatory protein (PhoB) and proteins known to be involved in regulation and transport functions (PstC, PstA, PhoB, PstS, PhoU) of inorganic P. The MPS clone F4-01 (Fig. 2C) has homology to P-associated reading frames to the sensor kinase (PhoR), regulatory protein (PhoB) and proteins involved in regulation and transport of phosphate (PhoU and Pst B). The MPS clone F42-01, (Fig. 2D) showed homology to a phosphate selective O and P porin. Porins associated with the outer membrane of gram-negative bacteria allow the diffusion of substrates between the external environment and the periplasm of the cell (Nikaido and Vaara 1985).The phosphate O and P porin are selective porins that have a higher affinity for phosphate and are well studied in *P aeruginosa* (Hancock et al. 1992; Siehnel et al. 1992). The porin O has a higher affinity for polyphosphates, while porin P has a higher affinity for orthophosphate and both are known to be expressed under P deficiency (Siehnel et al. 1992). MPS clone F41-01 and F40-01 (Fig. 2E and F) have a P-associated-like homolog PhoB, which is a major regulatory gene controlling the Pho regulon.

**Table 2:** Annotation of P-associated open reading frame (ORF) identified from mineral phosphate solubilization (MPS) clones/contigs from barley rhizosphere soil metagenome

MPS clone	Gene product	P-associated function in bacteria	Organism (best hit)	Accession no.	*E-*Value; Identities (*I*) (%)	Classified phylum/class
F39-01	*Pqq*	Cofactor involved in direct oxidation of glucose to gluconic acid (MPS activity)^1^	*Herpetosiphon aurantiacus* ATCC	YP_001546801.1	*E* = 4e-25; *I* = 77/274 (28%)	Chloroflexi
F25-01	*PhoB*	Inducer of Pho regulon^2^	*Sphaerobacter thermophilus* DSM	YP_003318630.1	*E* = 3E-73; *I* = 44/235 (61%)	Chloroflexi
F25-01	*PhoR*	Sensor protein of Pi^3^	*Thermobaculum terrenum*	YP_003324573.1	*E* = 1E-100; *I* = 203/418 (49%)	Unclassified bacteria
F25-01	*PstC*	Channel for Pi^4^	*Acidobacterium* sp. MP5ACTX9	YP_004217537.1	*E* = 4E-89; *I* = 77/317 (56%)	Acidobacteria
F25-01	*Pst A*	Channel for Pi^5^	*Acidobacterium* sp. MP5ACTX8	ZP_07032282.1	*E* = 3E-83; *I* = 54/271 (57%)	Acidobacteria
F25-01	*Pst B*	Component of transport system for Pi^6^	*Thermaerobacter marianensis* DSM	YP_004102482.1	*E* = 4E-92; *I* = 78/288 (62%)	Firmicutes
F25-01	*PhoU*	Modulator of Pi transduction^7^	*Dehalococcoides ethanogenes*	YP_180892.1	*E* = 7E-63; *I* = 22/219 (56%)	Chloroflexi
F25-01	*Pst S*	Pi-Binding protein^8^	*Soragium cellulosium*	YP_001613585.1	*E* = 7E-87; *I* = 168/360 (47%)	Deltaproteobacteria
F4-01	*Pst B*	Component of transport^6^	*Herbaspirillum seropedicae* SmR1	YP_003774682.1	*E* = 1E-85; *I* = 150/179 (84%)	Betaproteobacteria
F4-01	*PhoU*	Modulator of Pi transduction^7^	*Gallionella capsiferriformans* ES2	YP_003848266.1	*E* = 5E-63; *I* = 123/232 (53%)	Betaproteobacteria
F4-01	*PhoB*	Inducer of Pho regulon^2^	*Laribacter hongkongensis* HLHK9	YP_002794170.1	*E* = 4E-84; *I* = 146/226 (65%)	Betaproteobacteria
F4-01	*PhoR*	Sensor protein of Pi^3^	*Sideroxydans lithotrophicus* ES-1	YP_003522960.1	*E* = 3E-111; *I* = 202/418 (48%)	Betaproteobacteria
F42-01	*Phosphate –*selective *porin O/P*	Channel of Pi^9^	*Coraliomargarita akajimensis* DSM	YP_003549806.1	*E* = 4E-23; *I* = 73/244 (30%)	Verrucomicrobia
F40-01	*PhoB*	Inducer of Pho regulon^2^	*Nitrosospira multiformis ATCC*	YP_411941.1	*E* = 2E-108; *I* = 228/596 (38%)	Betaproteobacteria
F41-01	*PhoB*	Inducer of Pho regulon^2^	*Nitrosospira multiformis* ATCC	YP_411941.1	*E* = 5E-108; *I* = 228/596 (38%)	Betaproteobacteria

1 Rodriguez and Fraga (1999); Liu et al. (1992).

2 Makino et al. (1986); Vershinina and Znamenskaya (2002); Wanner (1993).

3 Vershinina and Znamenskaya (2002); Wanner (1993).

4 Vershinina and Znamenskaya (2002).

5 Surin et al. (1985); Vershinina and Znamenskaya (2002).

6 Chan and Torriani (1996); Vershinina and Znamenskaya (2002).

7 Muda et al. (1992); Vershinina and Znamenskaya (2002).

8 Surin et al. (1985); Vershinina and Znamenskaya (2002).

9 Nikaido and Vaara (1985); Hancock et al. (1992); Siehnel et al. (1992).

## Discussion

The plant rhizosphere actively secretes a number of compounds including simple sugars for example, glucose that promote the growth of rhizobacteria, which are hypothesized to have higher MPS activity based on the utilization of these carbon sources. A functional metagenomics approach is considered useful to study non cultivable rhizosphere microbiology (Leveau 2007) and this report identified a number of barley rhizosphere metagenome fosmids that conferred on *E. coli* an MPS phenotype. MPS activity was measured by the amount of Pi liberated from mineral P broth and was found to vary depending on the fosmid clone tested. The MPS phenotype was also associated with a rapid pH reduction (acidification) of the medium and in most cases the production of varying amounts of GA. However, there was no obvious relationship between the final pH, GA and the amount of phosphate solubilized. GA biosynthesis by MPS bacteria is a widely recognized mechanism known to be involved in MPS activity from a number of cultured isolates (Fuhrer et al. 2005; Del-Castillo et al. 2007, 2008; De-Werra et al. 2009) but this is the first report of this trait being described from the rhizosphere soil metagenome. GA and 2-KGA are products of the oxidative pathway of glucose utilization and is predicted to be widespread in bacteria resident in the rhizosphere (Goldstein and Liu 1987; Gyaneshwar et al. 1999). However, the precise mechanism of MPS has been a subject of analysis for some time and is still a matter of curiosity (Rodriguez and Fraga 1999). The pyrrolquinoline quinone (PQQ)-dependent GCD present in *E. coli* has been activated when PQQ biosynthesis gene(s) from MPS organisms were expressed in this host (Liu et al. 1992; Babu-Khan et al. 1995; Kim et al. 1998; Khairnar et al. 2003). *E. coli* itself appears to lack a full PQQ biosynthetic pathway and does not produce PQQ (Duine 1991; Matsushita et al. 1997; Krishnaraj and Goldstein 2001) and although *E. coli* has a well-defined P uptake and P regulatory system (Vershinina and Znamenskaya 2002) it is not known as an MPS organism.

Sequence analysis of the MPS clones showed no obvious gene or gene clusters relating to described genes/mechanisms to MPS activity with the exception of clone F39-01, which had one ORF with homology to PQQ-binding proteins. The closest match of this ORF (*E-*value = 4e-25) with identity of 28% at protein level was to a protein kinase containing PQQ-domain homologue in *Herpetosiphon aurantiacus ATCC 23,779* from the phylum *Chloroflexi*. The homology-based searches are not accurate to define an entire contig as belonging to a particular phylum/class (see Table 2) because of the limitations of available sequences in the dataset (Genbank). However, this study shows that these “best matches” phyla/class (Table 2) are diverse with in many cases no close homology to known cultured or defined uncultured sequences. The sequencing analysis data suggest that there exist other genes or pathways responsible for conferring MPS activity in *E. coli*. Results from previous studies has shown a role for genes such as *gabY* in *P. cepacia* and plasmid pKG3791 from *S. marcescens,* which have no homology to known GA biosynthetic genes, but appear to regulate GA biosynthesis in *E. coli*.

This study identified a number of novel ORF/homologs to P-regulatory and transport genes (i.e., Pho regulon gene[s]) (see Table 2). The Pho regulon regulates phosphate transport in bacteria (Vershinina and Znamenskaya 2002) and is also known to regulate a number of other functions (Monds et al. 2001; Hsieh and Wanner 2010). Studies on MPS genes from organisms such as *Erwinia herbicola* and *S. marcescens* suggest regulatory links to the Pho regulon, which are induced based on availability of Pi (Goldstein and Liu 1987; Goldstein et al. 1989; Bagyaraj et al. 2000; Krishnaraj and Goldstein 2001). In conclusion, the known pathway for GA synthesis in *E. coli* requires PQQ, however, other studies (Babu-Khan et al. 1995; Krishnaraj and Goldstein 2001) have shown that GA production occurs in the presence of other genes with no homology to PQQ. *E. coli* may have an alternative pathway for PQQ biosynthesis that is activated under certain conditions (Krishnaraj and Goldstein 2001). This study does highlight the inherent limitations in using heterologous hosts for functional screening of metagenomic libraries.

A previous study by Browne et al. (2009) with MPS active fluorescent Pseudomonads (gamma-proteobacteria) isolated from the same site showed that this trait was associated with a specific phylogenetic group within the fluorescent pseudomonas complex and Meyer et al. (2011) identified the *pqqC* gene as a useful molecular marker for studying the diversity and phylogeny of MPS pseudomonads. It is interesting that our metagenome approach identified novel systems (mechanistic and phylogenetic) with no phylogenetic relationship with the gamma-proteobacteria (Table 2). This study describes the first taxa and potential genes of the noncultivable MPS bacterial microbiome in barley rhizosphere soil and future work on these MPS clones will focus on understanding the potentially novel MPS mechanisms isolated in this study.
